# Recording single- and multi-unit neuronal action potentials from the surface of the dorsal root ganglion

**DOI:** 10.1038/s41598-019-38924-w

**Published:** 2019-02-26

**Authors:** Ahmed I. Kashkoush, Robert A. Gaunt, Lee E. Fisher, Tim M. Bruns, Douglas J. Weber

**Affiliations:** 1 Department of Physical Medicine and Rehabilitation, University of Pittsburgh, Pittsburgh, Pennsylvania United States of America; 2 Department of Bioengineering, University of Pittsburgh, Pittsburgh, Pennsylvania United States of America; 3 Center for the Neural Basis of Cognition, Pittsburgh, Pennsylvania United States of America; 4 Department of Biomedical Engineering, University of Michigan, Ann Arbor, Michigan United States of America; 5 Biointerfaces Institute, University of Michigan, Ann Arbor, Michigan United States of America

## Abstract

The dorsal root ganglia (DRG) contain cell bodies of primary afferent neurons, which are frequently studied by recording extracellularly with penetrating microelectrodes inserted into the DRG. We aimed to isolate single- and multi-unit activity from primary afferents in the lumbar DRG using non-penetrating electrode arrays and to characterize the relationship of that activity with limb position and movement. The left sixth and seventh lumbar DRG (L6-L7) were instrumented with penetrating and non-penetrating electrode arrays to record neural activity during passive hindlimb movement in 7 anesthetized cats. We found that the non-penetrating arrays could record both multi-unit and well-isolated single-unit activity from the surface of the DRG, although with smaller signal to noise ratios (SNRs) compared to penetrating electrodes. Across all recorded units, the median SNR was 1.1 for non-penetrating electrodes and 1.6 for penetrating electrodes. Although the non-penetrating arrays were not anchored to the DRG or surrounding tissues, the spike amplitudes did not change (<1% change from baseline spike amplitude) when the limb was moved passively over a limited range of motion (~20 degrees at the hip). Units of various sensory fiber types were recorded, with 20% of units identified as primary muscle spindles, 37% as secondary muscle spindles, and 24% as cutaneous afferents. Our study suggests that non-penetrating electrode arrays can record modulated single- and multi-unit neural activity of various sensory fiber types from the DRG surface.

## Introduction

Since the pioneering studies of Sir Edgar Adrian^1,2^, neuroscientists have been measuring the bioelectrical activity of primary afferent neurons to understand the neural code for touch^3–5^, proprioception^6,7^, and other sensory systems. In the somatosensory system, several approaches have been utilized to measure activity in peripheral nerves of anesthetized and behaving animals. Cuff electrodes can be placed around nerves to measure the electrical signals generated by action potentials propagating along the axons inside the nerve^8^. Intraneural electrodes penetrate the epineurium and measure these same action potentials inside the nerve^9^, while intrafascicular electrodes (a subset of intraneural electrodes), penetrate the perineurium and are positioned in direct proximity with the axons of the peripheral nerve^10^. The interested reader is referred to a review by Ortiz-Catalan *et al*.^11^, which provides an excellent overview of the various types of electrodes used to measure and stimulate activity in peripheral nerves.

While recording from peripheral nerves is possible, the dorsal root ganglion (DRG) presents an appealing target for measuring the activity of primary afferent neurons. The DRG contain the cell bodies and axons of passage exclusively of sensory neurons; the axons of motor neurons are located in the ventral roots and are thus isolated from the sensory neurons. The cell bodies of DRG neurons are generally very large and generate extracellular potentials that are easy to detect. Typically, DRG cell bodies are 40–50 μm in diameter, and can range from 5–110 μm^12^. Loeb, *et al*. and Prochazka, *et al*. were the first to demonstrate single neuron action potential recordings from DRG of freely moving cats, providing the first measurements of muscle and cutaneous afferent activity during normal locomotion^13,14^. Since those experiments, high-density arrays of penetrating microelectrodes have been used to measure the activity of tens to hundreds of neurons in parallel. For instance, Utah Electrode Arrays (UEAs) have been utilized to record simultaneously from large numbers (100 + ) of DRG neurons in anesthetized cats to examine correlations between limb-kinematics and the firing rates of muscle and cutaneous afferent neurons^15^. Simultaneous recordings from dozens of DRG neurons has also been achieved in awake cats using UEAs implanted chronically in the 7^th^ lumbar (L7) DRG, demonstrating limb-state decoding from ensembles of afferent neurons during locomotion^16^.

Previous studies have been limited to recording DRG activity via penetrating electrodes, which offer high spatial resolution, allowing for extracellular recording of action potentials from individual neurons distributed throughout the DRG. However, maintaining high-quality single-unit recordings from chronically implanted devices can be a challenge, as penetrating electrodes induce bleeding, inflammation, and gliosis, thus degrading recording quality over time^17–21^. While recordings with multielectrode arrays can be maintained in the cortex for years^1,2^, similar stability has been more difficult to achieve in the periphery^9,17,21^, likely due in part to the greater mobility of peripheral and spinal nerves. Additionally, penetrating arrays in spinal roots to target DRG are subject to mechanical failure as electrodes can be damaged by contact with surrounding vertebral bone during the implantation procedure. For both long-term investigations of somatosensory function in experimental animals, and also potentially for neuroprosthetic applications in people, stable recordings from primary sensory afferents in the DRG are essential.

As an alternative to penetrating electrodes, microelectrode arrays on a conformable substrate could be attached to the surface of the DRG, eliminating tissue damage associated with penetration of the epineurium. Such an approach may provide a more stable interface for long-term neural recording by reducing tissue inflammation and damage caused by relative movement of the electrodes and tissue^22^. However, a potential shortcoming of non-penetrating arrays is diminished signal-to-noise ratio, since recording sites are further from the cell bodies. Prior studies examining non-penetrating arrays have included electroneurogram (ENG) recordings utilizing nerve cuff electrodes on peripheral nerves. Whole-nerve ENG recordings are generally low signal-to-noise ratio signals^23,24^, and are thus most useful for measuring downstream action potential propagation of central stimulation and nerve conduction velocity^25^. Other studies have focused on electrocorticography, which measures biopotentials at the surface of the brain corresponding to the summed electrical activity of many neurons in the superficial layers of the cerebral cortex^26–29^. For instance, Khodagholy *et al*. recently demonstrated the ability to record single units from superficial cortical layers of rodents and humans utilizing non-penetrating electrode arrays^30^. Measuring single-unit activity from deeper cortical layers, such as layers IV and V, with non-penetrating techniques is more challenging, however, as cell bodies are located 1–2 mm beneath the cortical surface^31^. Neurons in the DRG, on the other hand, are organized very differently than cortex, with the highest density of cell bodies located at the outer surface of the DRG^32^. Thus, electrodes placed on the surface of the DRG would be in close proximity to the most superficial cell bodies.

In a preliminary report, we have shown that it is possible to record the electrical activity of individual primary afferent neurons from the epineural surface of the DRG^33^. Here, we expand on these findings and also compare signal quality and unit modulation during hindlimb movement obtained with non-penetrating electrodes to those obtained with traditional penetrating recording techniques. We also examine the short-term recording stability of these non-penetrating arrays during limb movements and the classes of somatosensory afferents, such as primary muscle spindles, secondary muscle spindles, and cutaneous afferents that can be recorded.

We demonstrate that it is possible to record single- and multi-unit spiking activity from the epineural surface of the DRG. Although the SNR is generally ~30% lower than signals obtained with penetrating microelectrodes, about 6% of units had SNR greater than the median SNR of units from penetrating arrays. Spike amplitudes remained stable during passive movement of the hindlimb, but further development of the electrode technology and implantation methods will be required to ensure stable recordings in a freely moving animal. Furthermore, detailed studies of DRG morphology examining the thickness of the epineurium and the size and distribution of neurons^32^, especially near the surface, could be used to build mathematical models for simulating the electric fields produced at the DRG surface by neurons of various types. Such models could provide a useful tool for designing electrode array geometries and signal processing methods to improve SNR.

## Methods

In these experiments, we sought to record modulated single- and multi-unit spiking activity from the DRG surface using arrays of non-penetrating microelectrode during hindlimb movement. Experiments were performed in a total of 7 anesthetized cats. All experimental procedures were approved by the University of Pittsburgh Institutional Animal Care and Use Committee, and all experiments were performed in accordance with relevant guidelines and regulations.

### Anesthesia

Anesthesia was induced utilizing a ketamine-xylazine mixture and maintained with 1–2% isoflurane. Vital signs, including blood pressure, respiratory rate, oxygen saturation, body temperature and end-tidal CO_2_ were monitored continuously throughout the experiment, and were maintained within physiological limits. At the experiment’s conclusion, cats were euthanized with a 5 mg/kg dose of potassium chloride.

### Neural recording

**Figure 1:**
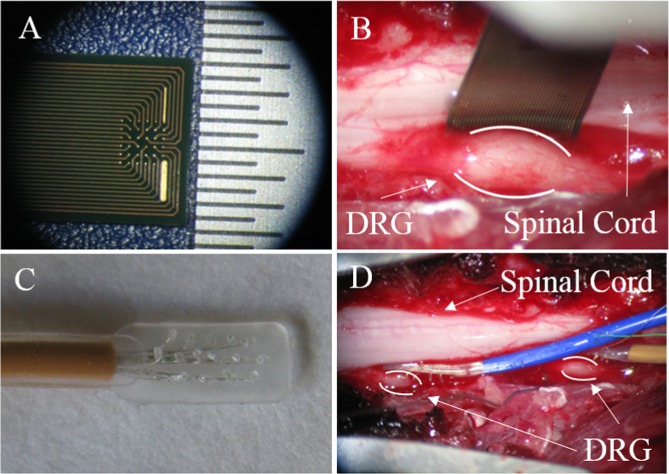
Images showing non-penetrating microelectrode arrays used in this study. (**A**) Close-up of 32-channel array manufactured by MCS. Hashes in scale bar separated by 500 µm. (**B**) MCS array shown placed on the surface of a lumbar DRG, between the DRG and the spinal cord. (**C**) Close-up of 16-channel array manufactured by PMT. Electrode sites separated by 1 mm. (**D**) PMT arrays shown placed on the surface of two DRG, between the DRG and the spinal cord.

After the animals were confirmed to be at a surgical plane of anesthesia, laminectomies were performed to expose the left sixth and seventh lumbar (L6-L7) DRG. Two different styles of non-penetrating electrode arrays (Fig. 1) were placed on the surface of both DRG: EcoFlexMEA36 (Multi Channel Systems Gmbh, Reutlinger, Germany) and Micro Electrode Arrays (PMT Corp., Chanhassen, MN), termed MCS and PMT arrays, respectively. The MCS array had 36 gold planar electrodes spaced 300 μm apart on a 6 × 6 polyimide square grid. The array contained a total of 32 recording electrodes, 2 internal reference electrodes, and 2 ground electrodes, which were all 50 μm in diameter. The PMT arrays were composed of 16 50-μm diameter platinum electrodes spaced 1 mm apart and arranged in 3 rows on a silicone rectangular grid.

We implanted 4 of the 7 cats with the MCS arrays (cats C, H, O, and P) and 2 cats with PMT arrays (cats H and R). We also implanted 40- and 50-channel Utah microelectrode arrays (1-mm shank length; 400 µm shank pitch; UEA; Blackrock Microsystems, Salt Lake City, UT) into the L6-L7 DRG in 5 cats (cats H, I, O, Q, R) to obtain neural recordings for a comparison group. Neural data were recorded using either an RZ2 (Tucker-Davis Technologies, Alachua, FL) or Grapevine (Ripple LLC, Salt Lake City, UT) multichannel neural recording system. The RZ2 sampling rate was 25 kHz and signals were filtered between 0.3–3 kHz. The sampling rate and filter bandwidth of the Grapevine system were 30 kHz and 0.25–7.5 kHz, respectively.

### Unit identification

In 3 trials in cat H, we administered intravenous succinylcholine chloride (SCh, 0.2 mg/kg) to identify muscle spindles, since SCh selectively increases the firing rate of primary and secondary muscle spindles (group Ia and II) afferents^34^. Units were classified as muscle spindles if their mean firing after SCh injection was at least 3 standard deviations above the mean firing rate before injection. Identified muscle spindle afferents were visually divided into group Ia and II afferents based on their static and dynamic responses. Units that modulated their firing rate based on muscle length, but not velocity, were classified as static spindle afferents (group II). Afferents with firing rates that were modulated by both muscle length and velocity, were classified as dynamic spindle afferents (group Ia). Cutaneous afferents were identified if they were modulated by limb perturbation but were not significantly activated by SCh injection. Golgi tendon organs (group Ib) tend to exhibit only a small, slow facilitation of firing under SCh^35^, but these neurons are typically inactive during passive leg motion due to their high activation threshold^36^, compounded by the loss of muscle tone induced by anesthesia.

### Passive limb movement experiments

We measured joint kinematics from the left hindlimb using 5 active markers positioned over the iliac crest, hip, knee, ankle, and metatarsal-phalangeal joint of each cat. Marker positions were recorded using a 6-camera motion tracking system (PhaseSpace Inc., San Fransisco, CA) at 120 Hz. The hindlimb was moved through a set of preprogrammed trajectories using an industrial robot (VS-6556E/GM, DENSO Robotics, Kariya, Aichi Prefecture, Japan) attached to the hindpaw. For each set of trials, the robot was programmed to perform either ramp-and-hold or center-out movements. For center-out trials, the robot moved the leg through 3- to 4.5-cm displacements at varying speeds in one of eight target directions: 0° (headward), 45°, 90° (up), 135°, 180°, 225°, 270° and 315° in the sagittal plane. The robot moved the leg sequentially to the 8 targets in a center-out pattern with 0.1 s dwell times at the center and each radial target. Movement velocities were varied between 7.4 and 29.5 cm/s. Ramp-and-hold movement trials were used to produce alternating flexion and extension movements of the whole leg. Here, the robot produced 4-cm displacement of the foot, with a 0.1-s dwell time at the flexed and extended positions, and the speed of the robot varied between 3.5 and 4.1 cm/s.

### Data analysis

Spike waveforms were detected by amplitude thresholding. Individual spikes were extracted from the filtered continuous data using a threshold crossing method where the threshold was set either using a fixed value or using a multiple of the signal standard deviation. Positive and negative thresholds were used, depending the shape of the waveform, but negative thresholds were used in the majority of cases. The median absolute value of the thresholds used in the non-penetrating electrodes was 15 μV (IQR, 12–19), while the median absolute value of thresholds for penetrating electrodes was 26 μV (IQR, 21–32). Spike waveforms (snippets) were extracted in 0.9–1.2 ms windows, spanning between 0.2–0.3 ms before and 0.7–0.9 ms after the threshold crossing point. The resulting spikes were sorted using OpenSorter (Tucker-Davis Technologies, Alachua, FL) and exported to MATLAB 2014a (Mathworks, Natick, MA) for further analysis.

Signal-to-noise ratio (SNR) was defined as the peak spike amplitude of the mean waveform for each sorted unit divided by three times the standard deviation of the background noise. For each unit, background noise was defined as the pre-spike baseline voltage, measured 160–300 μs before spikes. Peak-to-peak amplitude was calculated as the difference between the maximum and minimum voltage of the average waveform for each unit. Wavelength was defined as the time between the first peak and trough of the mean waveform. Interspike intervals (ISIs) were measured as the time between successive spikes. Sorted units were characterized as originating from a single neuron (single-unit activity) if less than 1% of ISIs were less than 2.5 ms, with all other recordings considered as originating from multiple neurons, labeled multi-unit activity. All sorted units were classified as either single or multi-units. Both single-unit and multi-unit waveforms were counted towards the total neuron population count.

Smoothed firing rates were calculated by convolving a 100-ms Gaussian kernel with the spike times for each unit. Multivariate regression models were used to examine the correlation between neural firing rate and two or more state variables measuring leg motion^16^. Joint angle and angular velocity of the hip, knee, and ankle served as the regressor variables and the smoothed firing rate time series for each unit served as the dependent variable. The regression models were fit to data from the center-out and ramp-and-hold trials. The coefficient of determination (R^2^) served as a measure of the correlation between firing rates and the weighted sum of the kinematic variables determined by a least-squares fit in the regression model.

### Statistics

We used a Wilcoxon rank-sum test to compare continuous variables (SNR, R^2^, units per electrode) between electrode types. Continuous variables are reported as median (interquartile range; IQR), unless otherwise indicated. A Chi-squared test, which is used to compare the proportions of categorical variables, was utilized to compare percent single-unit activity and percent of electrodes with recorded activity (dependent, categorical variables) between electrode types (independent, categorical variable). In cases where any of the values within the chi-squared contingency tables were less than 5, then we instead utilized a Fisher exact test, as the chi-squared test is not suitable in such cases. Differences in variables of interest were considered to be statistically significant if p < 0.05. All statistical analyses were performed utilizing MATLAB and SPSS v24 (IBM, Armonk, NY).

## Results

In this study, we observed that non-penetrating electrode arrays could record modulated, single-unit activity at the DRG surface. While SNR was generally lower than for units recorded by penetrating electrodes, it was possible to isolate many single units via non-penetrating recordings. Unit waveform amplitude was not sensitive to limb movement, suggesting that signal quality does not degrade appreciably over the limited range of motions that were tested. Furthermore, non-penetrating arrays were able to record action potentials from isolated neurons of different classes including primary and secondary muscle spindles as well as cutaneous afferents.

### Unit yield

Spike detection and sorting was performed on data from 26 trials, yielding an average (± standard deviation) of 25 ± 17 units in each trial. Trials with the highest unit count were used to estimate the yield for each electrode type. A total of 226 units (79 PMT in 2 trials, 147 MCS in 4 trials) were extracted from 6 non-penetrating trials. A total of 311 units were extracted from 5 trials with penetrating electrodes. On channels with recorded activity, there was a mean of 1.22 and 1.25 units recorded per channel on non-penetrating and penetrating arrays, respectively, which was not a statistically significant difference (p = 0.174, rank-sum test). The proportion of electrodes recording single-unit activity was similar between non-penetrating (44%) and penetrating arrays (45%) (p = 0.785, chi-squared test).

### SNR and unit modulation

**Figure 2:**
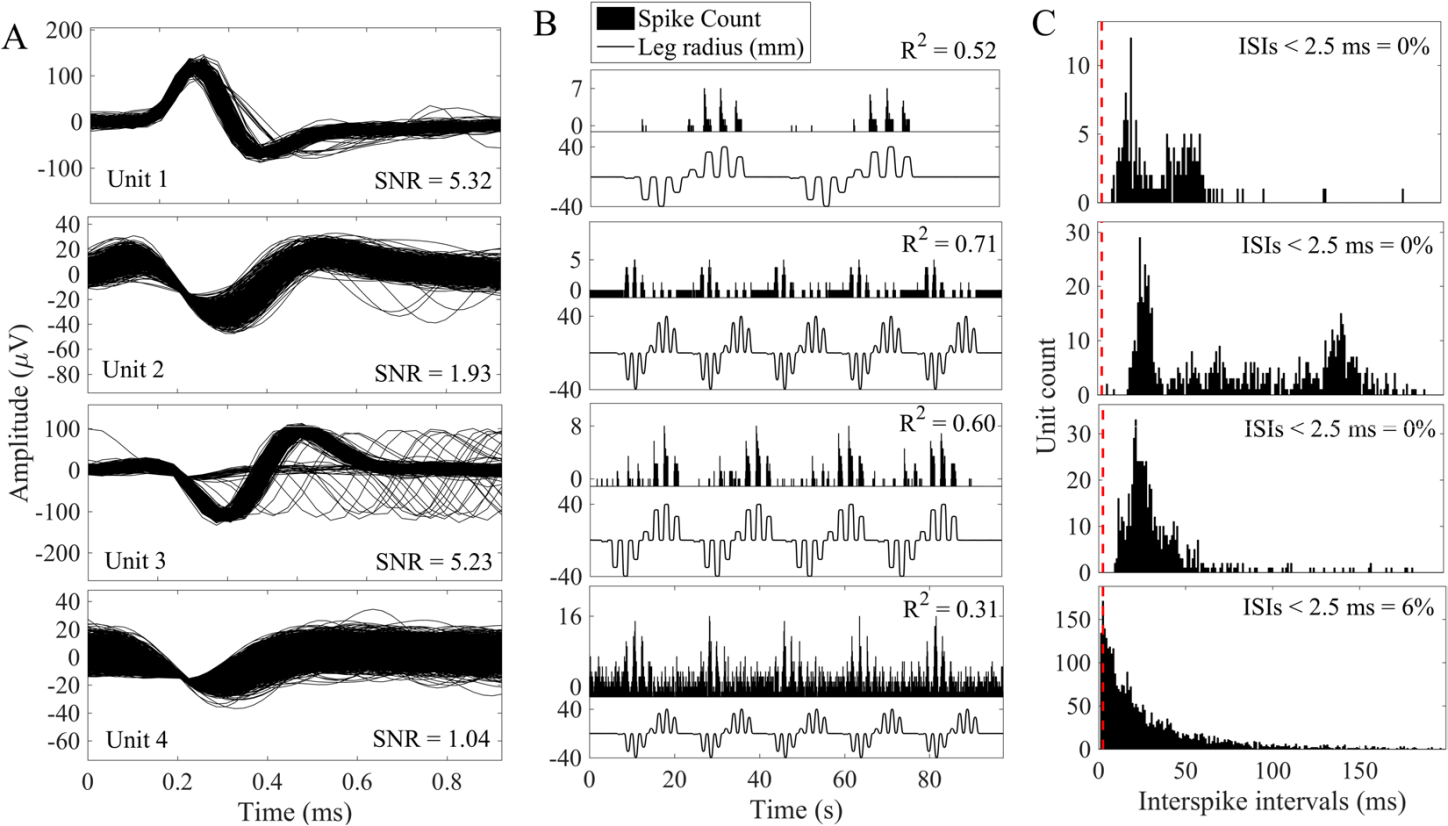
Example spike recordings from non-penetrating arrays. (**A**) Spike waveforms for isolated units on 1 penetrating electrode (Unit 1) and 3 different non-penetrating electrodes (Units 2–4). (**B**) Binned firing rates and corresponding limb kinematics from center-out motion trials. (**C**) ISI distributions for each unit. Units are classified as single unit recordings if < 1% of the ISIs are below 2.5 ms (dashed vertical line).

Figure 2 shows examples of various unit patterns obtained with penetrating and non-penetrating electrodes. Figure 2, Unit 1 is an exemplar unit from a penetrating electrode that has a high SNR ( > 5) and is modulated strongly during movement in some, but not all directions of the center-out pattern. Figure 2, Unit 2 shows an isolated single-unit with a moderate SNR recorded on a non-penetrating electrode and firing rate activity that is modulated strongly by limb movement. Figure 2, Unit 3 shows another single-unit from a non-penetrating electrode, with an SNR > 5 and firing rates that are strongly correlated with limb kinematics. Finally, Figure 2, Unit 4 shows an example of a unit that was classified as a multi-unit, because more than 1% of the ISIs were less than 2.5 ms. This multi-unit has a low SNR and was modulated weakly by limb movement.

**Figure 3:**
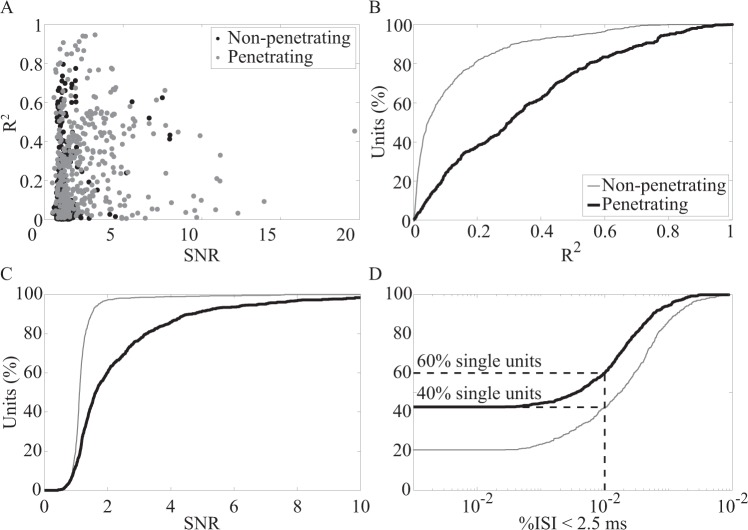
Comparison of signal quality for non-penetrating and penetrating electrode arrays. (**A**) Scatterplot of R^2^ vs SNR values for each unit. Cumulative distributions of (**B**) R^2^, (**C**) SNR, and (**D**) percentage of interspike intervals (ISI) less than 2.5 ms. SNR was calculated for all units recorded from non-penetrating (thick line; n = 655 units) and penetrating (thin gray line; n = 882 units) electrodes. R^2^ was calculated for all units for which limb kinematic data were collected (non-penetrating electrodes, n = 623 units; penetrating electrodes, n = 366).

Figure 3A shows a scatterplot of R^2^ values vs SNR for neurons recorded on penetrating and non-penetrating electrodes. Both electrode types recorded units having a wide range of SNR and R^2^ values, including many low-SNR neurons that exhibit no or very weak correlations (R^2^ < 0.2) with kinematic variables. However, it is interesting that there are several neurons with low SNR that exhibit fairly strong (R^2^ > 0.5) correlations with the kinematics. Conversely, there are several neurons with high SNR that are only weakly correlated with the leg motion.

The cumulative distributions for the R^2^ and SNR data are shown in panels B and C of Figure 3. R^2^ was found to be significantly different between units for the two array types (Fig. 3B). Median R^2^ was 0.05 (IQR, 0.02–0.15) for neurons from non-penetrating electrodes and 0.27 (IQR, 0.10–0.44) for neurons from penetrating electrodes (p < 0.0001, rank-sum test). The median SNR was 1.1 (IQR, 1.0–1.2) for units recorded from non-penetrating arrays and 1.6 (IQR, 1.2–2.8) for those recorded from penetrating arrays, which was a statistically significant difference (p < 0.0001, rank-sum test) (Fig. 3C).

**Figure 4:**
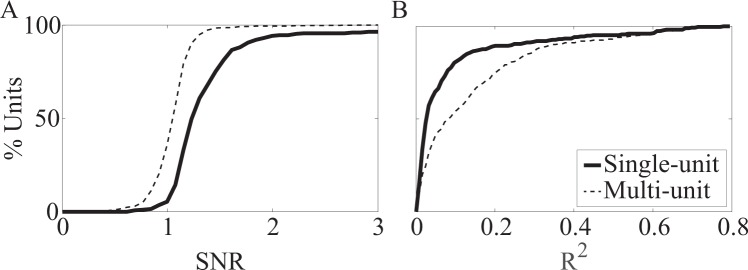
Comparison of signal quality for single- and multi-unit waveforms recorded on non-penetrating electrodes. Cumulative distributions of (**A**) SNR and (**B**) R^2^. SNR was calculated for all single-unit spikes (thick line, n = 278 units) and multi-unit spikes (thin dashed line, n = 377 units) during all trials. R^2^ was calculated for all trials where limb kinematics were collected (single-unit, n = 274 units; multi-unit, n = 347).

The R^2^ and SNR data shown in Figure 3A–C include the total set of single and multi-unit spikes recorded with both types of arrays. Units were classified as single units if < 1% of the total interspike intervals (ISIs) fell below 2.5 ms. Figure 3D shows the cumulative distribution of units based on the percentage of ISIs for each unit that were less than 2.5 ms. Single-unit activity composed 60% (529/882) and 42% (278/655) of all units obtained on penetrating and non-penetrating arrays, respectively. Of note, units classified as single units tended to have higher SNR and were more strongly correlated with limb kinematics than multi-unit activity (Fig. 4).

**Figure 5:**
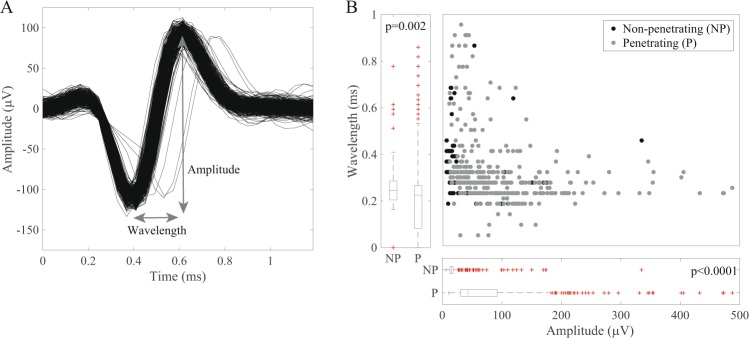
Waveform characteristics of units recorded from penetrating and non-penetrating electrodes. (**A**) Wavelength was defined as the time between the first peak and trough of the mean waveform. Amplitude was defined as the difference between the maximum and minimum voltage of the mean waveform. (**B**) Units recorded from non-penetrating (NP) electrodes tended to have longer wavelengths (p = 0.002, rank-sum test), whereas units from penetrating (P) electrodes tended to have larger amplitude (p < 0.0001, rank-sum test). Waveform amplitudes > 500 μV are not shown.

We utilized trials with the highest unit count to analyze if the amplitude and/or wavelengths differed between the two types of electrodes (n = 226 non-penetrating units, n = 311 penetrating units). We measured unit wavelength and peak-to-peak amplitude and compared distributions of these variables across array types (Fig. 5A). We observed that non-penetrating arrays were more likely to record units with higher wavelength (p = 0.002, rank-sum test) and that penetrating arrays recorded units had higher amplitude (p < 0.0001, rank-sum test) (Fig. 5B). Several factors may account for these differences. Penetrating electrodes have a higher impedance and are likely to be closer to the neurons being recorded, resulting in generally larger spike amplitudes. Non-penetrating electrodes are presumably further away from neurons and their signals are further attenuated by the epineurium, which is likely to have a higher impedance than the intraganglionic space. The differences in amplitude and wavelength may also be related to the types and distribution of cells, which may vary in size and density, throughout the DRG^32^.

Lastly, we compared unit characteristics between the MCS and PMT arrays to examine differences between the types of non-penetrating electrodes. Overall, units recorded on MCS arrays were more strongly correlated with the kinematics, whereas PMT arrays tended to record more single units and more units per electrode (Table 1). The SNR for the two electrode types was similar. Variations in electrode design and/or positioning on the DRG may have influenced the ability to record units.

**Table 1:** Summary of neural recordings obtained with MCS, PMT, and Utah arrays.

Variable	Array Type		
	MCS	PMT	Utah
Units	293	362	882
Single-unit activity	23% (66/293)	59% (212/362)	60% (529/882)
Units per electrode*	1.20 (1.17–1.23)	1.38 (1.34–1.42)	1.25 (1.21–1.29)
SNR	1.1 (1.0–1.2)	1.1 (1.0–1.3)	1.6 (1.2–2.8)
R^2^	0.12 (0.04–0.24)	0.02 (0.01–0.07)	0.27 (0.10–0.44)

Continuous variables are reported as median (IQR). R^2^-values indicate the coefficient of determination for the correlation between firing rates and kinematic variables as measured via multivariate linear regression.

^*^Reported as mean (95% CI).

### Effect of mechanical perturbation on signal quality

**Figure 6:**
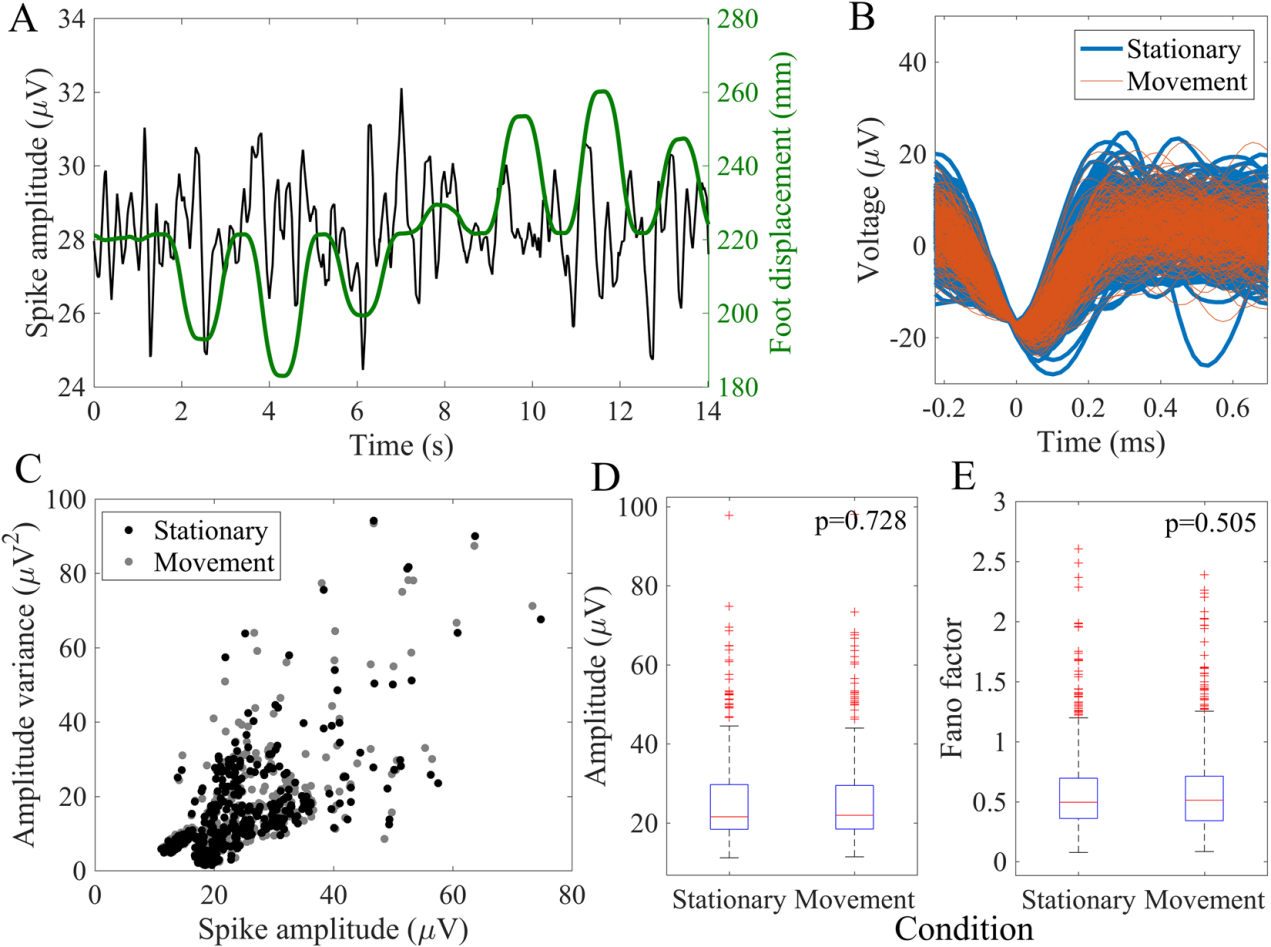
Effect of limb movement on unit spike amplitude. (**A**) Spike amplitude and foot displacement averaged over 5 center-out motion trials for an example single unit. (**B**) Example unit waveforms during epochs where the limb was stationary (blue) or moving (red). (**C**) Scatterplot of amplitude variance versus spike amplitude units (n = 474 units, 5 cats). (**D**) Distribution of spike amplitudes between stationary and movement phases (p = 0.728, rank-sum test). (**E**) Rank-sum test applied across all units showed no significant difference in amplitude Fano factors between conditions (p = 0.505). Fano factors > 3 and spike amplitudes > 100 μV not shown in figure.

Since the non-penetrating arrays were not mechanically anchored to tissue, we were concerned that the electrodes might shift on the surface of the DRG and therefore lead to poor recording stability. We measured spike amplitude during periods when the limb was stationary or moving passively during the ramp-and-hold and center-out trials. Figure 6A shows an overlay of the kinematics and neural signal amplitude from one electrode. Figure 6B shows spike waveforms for an exemplar unit during the stationary and movement periods. We extracted a subset of 474 units in which movement times and kinematic data were recorded simultaneously. For each unit, we compared the distribution of spike amplitudes during stationary and movement phases. The median difference in peak-to-peak amplitude across all units between conditions was 0.24 μV (interquartile range, 0.11–0.47), or ~1% of baseline spike amplitude. No significant differences were observed in median spike amplitude (p = 0.728, rank-sum test) between movement conditions across all units (Fig. 6D). No unit had greater than 10% variation in spike amplitude during motion from baseline spike amplitude. We further evaluated the Fano factor for each unit, which is the signal variance divided by its mean, for waveform amplitudes during the movement and stationary phases to observe if mechanical perturbation affected signal variability. The difference in Fano factors between movement conditions for waveform amplitude was non-significant (p = 0.505, rank-sum test) across all units (Fig. 6E).

### Diversity of recruited fiber types

**Figure 7:**
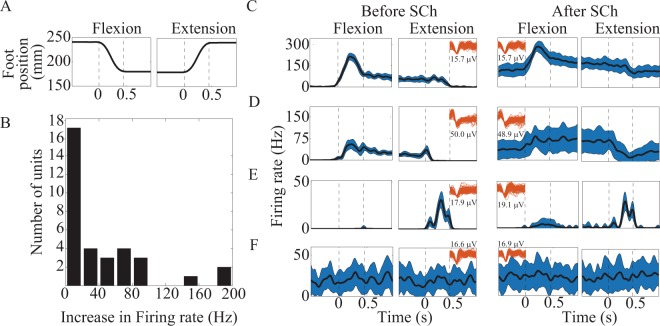
SCh injection for muscle spindle identification. (**A**) Foot displacement during the ramp-and-hold trial. (**B**) Histogram of firing rate changes after SCh injection for units classified as muscle spindle afferents. (**C**) A unit with strong SCh response that was differentially modulated by perturbation direction with a dynamic response during flexion of the leg (flexion phase bounded by dashed lines), likely representing a primary muscle spindle (type Ia) afferent. (**D**) A unit with modest SCh response with a small dynamic response during flexion of the leg, likely representing a secondary muscle spindle (type II) afferent. (**E**) A unit that responds during leg extension movements, but appears unaffected by SCh, likely representative of a cutaneous afferent. (**F**) A unit without response to SCh or limb motion. Firing rates are displayed as mean (line) ± standard deviation (shaded area). Unit waveforms (red, amplitude given below or above) displayed in (**C**–**F**) to show stability of signal amplitude before and after SCh injection.

Finally, we performed a total of 3 SCh trials in cat H to identify muscle spindle activity. Units that were present in multiple trials were only counted once towards the total count. A total of 59 units were identified during these trials (19.7 units per trial), and of these, 34 (58%) were found to have increased firing rate after SCh injection. Muscle spindles were further classified based on their response to muscle stretch in order to identify group Ia (dynamic + static response) and group II (static response only) afferents. Of the 34 neurons that increased their firing rates in response to SCh injection, 12 (35%) were identified as primary muscle spindles afferents and 22 (65%) as secondary muscle spindle afferents (Fig. 7C,D). Units identified as primary muscle spindles had a median increase in firing rate of 52 Hz (IQR, 35–85) after SCh injection, whereas secondary muscle spindles increased firing by a median of 3 Hz (IQR, 2–11). Fourteen total units (24%) were not activated by SCh but were modulated by limb motion and were therefore classified as cutaneous afferents (Fig. 7E). Eleven units (19%) were not modulated by SCh or limb motion (Fig. 7F). These units may have been poorly recorded neurons, threshold crossing events that were merely noise, or primary afferents with receptive fields on parts of the limb that were not disturbed during movement. Some neurons, especially Golgi tendon organs and joint receptors tend to have a high threshold for responding, especially in the anesthetized preparations used here. Furthermore, the range of motion at the hip was limited to ~20 degrees in these experiments, which may not have been sufficient to modulate activity in muscle and cutaneous afferents that have proximal receptive fields.

## Discussion

### Key results

This study demonstrates that non-penetrating electrodes can record single-unit activity from the surface of lumbar DRG that in many cases, is modulated by passive movement of the hindlimb. The SNR was generally lower for spike waveforms detected on non-penetrating electrodes, but approximately 6% of units from non-penetrating arrays had an SNR greater than the median SNR of units from penetrating arrays. Regardless of SNR, the firing rates for units recorded with both types of electrodes were modulated with passive movement of the hindlimb, although the strength of correlation varied widely across neurons. Thus, the firing rates of DRG neurons recorded with non-penetrating electrodes may provide information that would be useful for estimating limb kinematics, since as few as ten primary afferent neurons may be sufficient to adequately estimate limb position using multivariate linear regression models^37^.

Our study also provides electrophysiological evidence that the cell bodies near the DRG surface are composed of various functional classes of primary afferents, including muscle spindles and mechanosensitive cutaneous afferents. Recording from a variety of fiber types is crucial to understanding how proprioceptive and tactile inputs contribute to neural representations of limb-state. For instance, multiple studies have demonstrated that encoding limb position and movement in multi-articular muscles require the input of both cutaneous and spindle afferents^16,38,39^. Previous studies on postural control have shown that both cutaneous and muscle afferents assist in maintaining balance^40,41^. The diverse neural data gathered by non-penetrating electrodes may allow for detailed study regarding interactions between neuronal subtypes underlying sensory integration and closed-loop control. For instance, non-penetrating arrays may be utilized in conjunction with penetrating arrays to record simultaneously from deep and superficial layers of the DRG simultaneously. Such a setup could significantly increase population yield and could also provide insights on spatial and temporal relationships between DRG populations.

We suspect that the single-unit activity recorded in our study can likely be regarded as a result of the cell bodies located directly beneath the epineurium. A study which examined neuronal activity from hippocampal CA1 pyramidal cells (average soma volume 2,066 μm^3^) and interneurons in anesthetized rats, estimated that extracellular spiking activity could be recorded up to 140 μm from the tetrode tip, which approximates cat epineural thickness (20–100 μm)^32,42,43^. Additionally, recent work from the lab of co-author (Bruns) used non-penetrating arrays of densely packed electrodes (25 μm pitch) to record neural activity from the surface of lumbosacral DRG in cats and estimated the location of some neural sources to be within 107 μm below the DRG surface^44^. Moreover, computational studies demonstrate that cell bodies within the DRG are organized non-randomly in clusters, with the majority of units located dorsally and in the outer 24% radially^32^. Histological study of the DRG further demonstrates that sensory neurons are organized in longitudinal bands around the perimeter of the DRG^45^. Thus, penetrating electrodes that record from the inner depths of the DRG may not be ideally suited to accommodate the circumferential organization of the DRG. Electrodes placed on or just below the DRG surface may be in closer proximity to primary afferent somata than electrodes placed near the core. Furthermore, neuronal recordings from the DRG surface may be biased towards recordings from the largest cell bodies, such as type Ia afferents that encode limb movement, as these units generally produce large-amplitude action potentials. In our analyses, we did observe that some high amplitude units (SNR > 4) were very closely related to limb kinematics, although there were too few (n = 7) to warrant further sub-group analysis.

### Challenges and potential improvements

A significant challenge to long-term recording capability is that non-penetrating electrodes, such as those utilized in this study, may be susceptible to motion artifact or drift if the arrays are not anchored securely to the tissue. In this study, the arrays were placed on the DRG surface without suturing or adhesives. Motion artifacts may be less problematic in the DRG, which are relatively immobile and surrounded by bony vertebrae, but a method for anchoring the electrode array to the tissue will be required. Biocompatible adhesive hydrogels may be used as electrode substrates or coatings to provide strong attachment to the tissue^46^.

Ideally, arrays should be flexible and thin to conform to an irregular surface, as the DRG is curved and ellipsoid-shaped. Moreover, the electrodes should be low-impedance to maximize SNR and could be printed on bioresorbable adhesive substrates in order to promote long-term stability at the tissue-electrode interface without slippage^30,33,47^. The non-penetrating electrode arrays used in this study were adapted from ones used primarily for neuronal cell cultures (MCS) or micro-electrocorticography (PMT), and neither are well-suited for DRG applications. Despite this limitation, neural waveform quality was not degraded during passive motion of the leg, which mimics at least a portion of the mechanical perturbations that may be present during walking.

Innovations in the design, fabrication methods, and materials used to build electrodes may lead to improvements in yield and long-term reliability. For example, transfer printing of electrode arrays to adhesion-promoting hydrogel substrates can potentially improve tissue integration and mechanical stability^48^. Another example, NeuroGrid is a high-density, low-impedance non-penetrating electrode array that was designed to conform to the curvilinear surface of the brain, and was observed to record single-unit activity for up to 10 days in humans and freely moving rats without device failure^30^. Other groups are investigating flexible polyimide microelectrode arrays that can conform to the surface of DRG^44^. Similar to NeuroGrid arrays implanted in the cortex^30^, non-penetrating electrode arrays could be adapted to chronically record single-unit activity from the DRG surface.

There is mounting evidence from studies of penetrating microelectrodes that reducing the surface area of the electrode helps to reduce the size of the foreign body response^49^. This would suggest that DRG surface arrays should be designed as an open mesh, rather than a continuous sheet to reduce exposure of tissue to foreign materials. Further innovation in electrode design may allow for long-term recording studies that characterize host reactions adjacent to the electrode and signal quality stability over time. Innovations such as changes in electrode geometry and materials may be used to reduce electrode impedance and improve isolation of single unit activity through use of polytrodes and source localization methods^44,50^.

### Research and clinical applications

Non-penetrating electrodes may offer a number of potential advantages over penetrating electrodes arrays related to the surgical aspects of device implantation, neural tissue damage, chronic foreign body responses, and device longevity. Non-penetrating arrays would be expected to induce less tissue damage around the contact sites, which may improve long-term stability of the neural interface^22^. Furthermore, non-penetrating arrays may be made of thinner and more flexible materials than penetrating electrodes, which require a stiff substrate for insertion. To that effect, non-penetrating electrodes have been shown to stimulate and stably recruit neural populations for over a year in nerves in both upper and lower limbs in humans^22,51^.

Furthermore, there are several potential clinical applications for neuronal recordings using non-penetrating electrode arrays. Sensory neural recordings from sacral DRG could be used in closed-loop neuroprosthetics for conditions such as bladder dysfunction in patients with spinal cord injury. Current bladder neuroprostheses are unidirectional systems that utilize sacral spinal root stimulation for bladder voiding, but do not convey information about bladder fullness^52^. Recording single-unit activity from bladder mechanosensitive afferents from the epineural surface of the DRG, may enable measurement of bladder pressure^21,53–57^, which may in turn improve the functionality of bladder control devices. Accordingly, recording from sacral DRG during changes in bladder pressure has recently been demonstrated with non-penetrating electrodes^44^.

Another potential application for this approach is the sensory augmentation of prosthetic limbs. DRG are attractive implantation sites for a somatosensory neural interface since they are anatomically isolated from motor neurons, can be accessed utilizing minimally invasive approaches, house cell bodies from multiple peripheral nerves within a single ganglion, and demonstrate a dermatomal rostrocaudal somatotopy^45,58,59^. Cervical and lumbar DRG recordings from human subjects may be utilized to better inform the design of biomimetic stimulation patterns for sensory restoration in patients with limb amputation^60^ or to provide limb-state feedback for closed-loop functional electrical stimulation^37,61^. Additionally, from an operative perspective, non-penetrating arrays offer size and compliance advantages not afforded to traditional electrode arrays. Non-penetrating electrodes can be customized for percutaneous implantation^62^, which removes the need for invasive surgical procedures that are required for penetrating array implants.

### Limitations

Several factors may have limited our study’s results. Our study was limited by the mechanical and electrical properties of the non-penetrating arrays utilized to record primary afferent activity. Increasing the compliance of the array surface and reducing the impedance of electrode contacts may improve the electrode-tissue interface and improve signal quality^47,48,63,64^. Also, thresholds for spike detection were generally set lower for non-penetrating arrays compared to those from penetrating arrays, which may have allowed for a preponderance of low-SNR units to be recorded from non-penetrating arrays.

We were unable to identify the precise location of neurons recorded in the DRG, and we did not always identify the specific cell type for each of the recorded neurons. Thus, we cannot draw strong conclusions about the selectivity of non-penetrating electrodes for recording specific cell types or their proximity to the electrodes. Single microelectrode recording methods tend to be biased toward detecting larger cells, while stereotrode and tetrode configurations offer better capabilities for isolating spike waveforms from smaller or more densely packed neurons. We expect that non-penetrating electrodes will show a similar and perhaps stronger bias, given the greater distance between neuron and electrode and the higher tissue impedance posed by the epineurium.

We compared DRG recordings from penetrating and non-penetrating electrodes obtained in separate experiments. Recording simultaneously from both electrode types positioned in the same area would have allowed for more direct comparisons of signal properties. Multisite penetrating microelectrodes would enable recording at multiple depths, which combined with surface recordings, might provide insight into the relationship between neuronal depth and surface signal characteristics.

The experiments used to evaluate stability of the neural signals in the presence of hindlimb movement were performed under passive conditions and over a limited range of motion. The specific manipulations (e.g. center-out, ramp-and-hold displacements) are identical to ones we have used in previous studies examining coding properties of DRG neurons^65^. Under these conditions, the angular displacements at the hip and knee were approximately 20 and 45 degrees, respectively. During feline locomotion, the hip undergoes approximately 50 degrees of rotation, while the range of motion for the knee is only about 30 degrees (see Fig. 3 in^16^). Further experiments would be necessary to evaluate the stability of surface electrodes over larger movements, and importantly, in chronic implants in behaving animals.

Several challenges remain in transitioning this approach to chronic implants, especially in larger animals and humans. It is possible that encapsulation occurs up to scales of 100 microns or more in the chronic setting, which would lead to smaller amplitudes and signal loss. It is also reasonable to assume that the epineurium in humans will be thicker than in cats, which will likely lead to smaller SNR. Non-penetrating electrodes should cause less or no damage to neural tissue, which may result in improving the long-term stability of the neural interface, but other issues related to scar tissue forming around the array will likely be similar to penetrating electrodes. Furthermore, encapsulation of the electrode array by connective tissue would further degrade SNR, as is seen with penetrating microelectrodes. Thus, transitioning this approach to long-term use will require that electrode arrays be designed to ensure tight adhesion to the DRG surface, while minimizing the formation of connective tissue, especially between the electrode array and DRG.

## Conclusions

Non-penetrating electrode arrays can record isolated single-unit neural activity from the DRG surface. Although units recorded on penetrating electrodes had generally higher SNR, non-penetrating arrays could record from many neurons with sufficient SNR to isolate individual units. Signal quality recorded from the DRG surface was not degraded during trials that included passive movement of the limb, with minimal change in waveform amplitude, although the range of motion for the hindlimb was limited in these experiments. Furthermore, recordings from non-penetrating electrodes could isolate primary afferents of different fiber types such as muscle spindles and cutaneous afferents. These results suggest that a non-penetrating DRG electrode interface may be a viable option for recording signals from primary somatosensory afferents for both basic science and neuroprosthetic applications.

## Data Availability

The datasets described in this study are available upon request from the corresponding author.

## References

[1] Adrian ED (1926). The impulses produced by sensory nerve endings: Part I. J Physiol.

[2] Adrian ED, Cattell M, Hoagland H (1931). Sensory discharges in single cutaneous nerve fibres. J Physiol.

[3] Adrian ED, Zotterman Y (1926). The impulses produced by sensory nerve endings: Part 3. Impulses set up by Touch and Pressure. J Physiol.

[4] Horch KW, Whitehorn D, Burgess PR (1974). Impulse generation in type I cutaneous mechanoreceptors. J Neurophysiol.

[5] Horch KW, Tuckett RP, Burgess PR (1977). A key to the classification of cutaneous mechanoreceptors. J Invest Dermatol.

[6] Stein RB (2004). Coding of position by simultaneously recorded sensory neurones in the cat dorsal root ganglion. J Physiol.

[7] Prochazka A (1986). Proprioception during voluntary movement. Can J Physiol Pharmacol.

[8] Struijk JJ, Thomsen M, Larsen JO, Sinkjaer T (1999). Cuff electrodes for long-term recording of natural sensory information. IEEE Eng Med Biol Mag.

[9] Branner A, Stein RB, Fernandez E, Aoyagi Y, Normann RA (2004). Long-term stimulation and recording with a penetrating microelectrode array in cat sciatic nerve. IEEE Trans Biomed Eng.

[10] Lawrence SM, Dhillon GS, Jensen W, Yoshida K, Horch KW (2004). Acute peripheral nerve recording characteristics of polymer-based longitudinal intrafascicular electrodes. IEEE Trans Neural Syst Rehabil Eng.

[11] Ortiz-Catalan M, Branemark R, Hakansson B, Delbeke J (2012). On the viability of implantable electrodes for the natural control of artificial limbs: review and discussion. Biomed Eng Online.

[12] Ambrose WW, McNeill ME (1978). Graphic representation of the distribution of acetylcholinesterase in cat dorsal root ganglion neurons. Histochem J.

[13] Loeb GE, Bak MJ, Duysens J (1977). Long-term unit recording from somatosensory neurons in the spinal ganglia of the freely walking cat. Science.

[14] Prochazka A, Westerman RA, Ziccone SP (1976). Discharges of single hindlimb afferents in the freely moving cat. J Neurophysiol.

[15] Aoyagi Y, Stein RB, Branner A, Pearson KG, Normann RA (2003). Capabilities of a penetrating microelectrode array for recording single units in dorsal root ganglia of the cat. J Neurosci Methods.

[16] Weber DJ, Stein RB, Everaert DG, Prochazka A (2007). Limb-state feedback from ensembles of simultaneously recorded dorsal root ganglion neurons. J Neural Eng.

[17] Debnath S, Bauman MJ, Fisher LE, Weber DJ, Gaunt RA (2014). Microelectrode array recordings from the ventral roots in chronically implanted cats. Front Neurol.

[18] Schmidt S, Horch K, Normann R (1993). Biocompatibility of silicon-based electrode arrays implanted in feline cortical tissue. J Biomed Mater Res.

[19] Turner JN (1999). Cerebral astrocyte response to micromachined silicon implants. Exp Neurol.

[20] Winslow BD, Tresco PA (2010). Quantitative analysis of the tissue response to chronically implanted microwire electrodes in rat cortex. Biomaterials.

[21] Khurram A (2017). Chronic monitoring of lower urinary tract activity via a sacral dorsal root ganglia interface. J Neural Eng.

[22] Fisher LE, Tyler DJ, Anderson JS, Triolo RJ (2009). Chronic stability and selectivity of four-contact spiral nerve-cuff electrodes in stimulating the human femoral nerve. J Neural Eng.

[23] Jezernik S, Wen JG, Rijkhoff NJ, Djurhuus JC, Sinkjaer T (2000). Analysis of bladder related nerve cuff electrode recordings from preganglionic pelvic nerve and sacral roots in pigs. J Urol.

[24] Kurstjens GA, Borau A, Rodriguez A, Rijkhoff NJ, Sinkjaer T (2005). Intraoperative recording of electroneurographic signals from cuff electrodes on extradural sacral roots in spinal cord injured patients. J Urol.

[25] Ayers CA, Fisher LE, Gaunt RA, Weber DJ (2016). Microstimulation of the lumbar DRG recruits primary afferent neurons in localized regions of lower limb. J Neurophysiol.

[26] Kaiju T (2017). High Spatiotemporal Resolution ECoG Recording of Somatosensory Evoked Potentials with Flexible Micro-Electrode Arrays. Front Neural Circuits.

[27] Wang W (2013). An electrocorticographic brain interface in an individual with tetraplegia. PLoS One.

[28] Wang W (2009). Human motor cortical activity recorded with Micro-ECoG electrodes, during individual finger movements. Conf Proc IEEE Eng Med Biol Soc.

[29] Buzsaki G, Anastassiou CA, Koch C (2012). The origin of extracellular fields and currents–EEG, ECoG, LFP and spikes. Nat Rev Neurosci.

[30] Khodagholy D (2015). NeuroGrid: recording action potentials from the surface of the brain. Nat Neurosci.

[31] Asanuma H, Rosen I (1972). Topographical organization of cortical efferent zones projecting to distal forelimb muscles in the monkey. Exp Brain Res.

[32] Ostrowski AK, Sperry ZJ, Kulik G, Bruns TM (2017). Quantitative models of feline lumbosacral dorsal root ganglia neuronal cell density. J Neurosci Methods.

[33] Gaunt RA (2011). Single- and multi-unit activity recorded from the surface of the dorsal root ganglia with non-penetrating electrode arrays. Conf Proc IEEE Eng Med Biol Soc.

[34] Fehr HU (1965). Activation by Suxamethonium of Primary and Secondary Endings of the Same De-Efferented Muscle Spindle during Static Stretch. J Physiol.

[35] Dutia MB, Ferrell WR (1980). The effect of suxamethonium on the response to stretch of Golgi tendon organs in the cat. J Physiol.

[36] Houk JC, Singer JJ, Henneman E (1971). Adequate stimulus for tendon organs with observations on mechanics of ankle joint. J Neurophysiol.

[37] Bruns TM, Wagenaar JB, Bauman MJ, Gaunt RA, Weber DJ (2013). Real-time control of hind limb functional electrical stimulation using feedback from dorsal root ganglia recordings. J Neural Eng.

[38] Biggs J, Horch K, Clark FJ (1999). Extrinsic muscles of the hand signal fingertip location more precisely than they signal the angles of individual finger joints. Exp Brain Res.

[39] Collins DF, Refshauge KM, Gandevia SC (2000). Sensory integration in the perception of movements at the human metacarpophalangeal joint. J Physiol.

[40] Honeycutt CF, Nardelli P, Cope TC, Nichols TR (2012). Muscle spindle responses to horizontal support surface perturbation in the anesthetized cat: insights into the role of autogenic feedback in whole body postural control. J Neurophysiol.

[41] Bloem BR, Allum JH, Carpenter MG, Honegger F (2000). Is lower leg proprioception essential for triggering human automatic postural responses?. Exp Brain Res.

[42] Henze DA (2000). Intracellular features predicted by extracellular recordings in the hippocampus *in vivo*. J Neurophysiol.

[43] Altemus KL, Lavenex P, Ishizuka N, Amaral DG (2005). Morphological characteristics and electrophysiological properties of CA1 pyramidal neurons in macaque monkeys. Neuroscience.

[44] Sperry ZJ (2018). Flexible microelectrode array for interfacing with the surface of neural ganglia. J Neural Eng.

[45] Wessels WJ, Feirabend HK, Marani E (1994). The rostrocaudal organization in the dorsal root ganglia of the rat: a consequence of plexus formation?. Anat Embryol (Berl).

[46] Huang W-C (2018). Ultracompliant Hydrogel-Based Neural Interfaces Fabricated by Aqueous-Phase Microtransfer Printing. Advanced Functional Materials.

[47] Kim DH (2010). Dissolvable films of silk fibroin for ultrathin conformal bio-integrated electronics. Nat Mater.

[48] Wu H (2015). Transfer Printing of Metallic Microstructures on Adhesion-Promoting Hydrogel Substrates. Advanced Materials.

[49] Seymour JP, Kipke DR (2007). Neural probe design for reduced tissue encapsulation in CNS. Biomaterials.

[50] Wodlinger B, Durand DM (2009). Localization and recovery of peripheral neural sources with beamforming algorithms. IEEE Trans Neural Syst Rehabil Eng.

[51] Polasek KH, Hoyen HA, Keith MW, Kirsch RF, Tyler DJ (2009). Stimulation stability and selectivity of chronically implanted multicontact nerve cuff electrodes in the human upper extremity. IEEE Trans Neural Syst Rehabil Eng.

[52] Bruns TM, Gaunt RA, Weber DJ (2011). Multielectrode array recordings of bladder and perineal primary afferent activity from the sacral dorsal root ganglia. J Neural Eng.

[53] Bahns E, Halsband U, Janig W (1987). Responses of sacral visceral afferents from the lower urinary tract, colon and anus to mechanical stimulation. Pflugers Arch.

[54] Habler HJ, Janig W, Koltzenburg M (1993). Myelinated primary afferents of the sacral spinal cord responding to slow filling and distension of the cat urinary bladder. J Physiol.

[55] Iggo A (1955). Tension receptors in the stomach and the urinary bladder. J Physiol.

[56] Winter DL (1971). Receptor characteristics and conduction velocites in bladder afferents. J Psychiatr Res.

[57] Ross SE, Ouyang Z, Rajagopalan S, Bruns TM (2018). Evaluation of Decoding Algorithms for Estimating Bladder Pressure from Dorsal Root Ganglia Neural Recordings. Ann Biomed Eng.

[58] Wessels WJ, Feirabend HK, Marani E (1990). Evidence for a rostrocaudal organization in dorsal root ganglia during development as demonstrated by intra-uterine WGA-HRP injections into the hindlimb of rat fetuses. Brain Res Dev Brain Res.

[59] Wessels WJ, Marani E (1993). A rostrocaudal somatotopic organization in the brachial dorsal root ganglia of neonatal rats. Clin Neurol Neurosurg.

[60] Weber DJ (2011). Limb-state information encoded by peripheral and central somatosensory neurons: implications for an afferent interface. IEEE Trans Neural Syst Rehabil Eng.

[61] Holinski BJ, Everaert DG, Mushahwar VK, Stein RB (2013). Real-time control of walking using recordings from dorsal root ganglia. J Neural Eng.

[62] Deer TR, Grigsby E, Weiner RL, Wilcosky B, Kramer JM (2013). A Prospective Study of Dorsal Root Ganglion Stimulation for the Relief of Chronic Pain. Neuromodulation: Technology at the Neural Interface.

[63] Venkatraman S (2011). *In vitro* and *in vivo* evaluation of PEDOT microelectrodes for neural stimulation and recording. IEEE Trans Neural Syst Rehabil Eng.

[64] Xiao Y, Martin DC, Cui X, Shenai M (2006). Surface modification of neural probes with conducting polymer poly(hydroxymethylated-3,4- ethylenedioxythiophene) and its biocompatibility. Appl Biochem Biotechnol.

[65] Wagenaar JB, Ventura V, Weber DJ (2011). State-space decoding of primary afferent neuron firing rates. J Neural Eng.

